# Battlefield Neck Injuries: Contemporary Insights From the Israeli National Trauma Registry

**DOI:** 10.1016/j.acepjo.2025.100211

**Published:** 2025-06-23

**Authors:** Nir Tsur, Dean Dudkiewicz, Tomer Talmy, Irina Radomislensky, Adi Givon, Eldad Katorza, Israel Trauma Group, H. Bahouth, M. Bala, A. Bar, A. Braslavsky, D. Czeiger, D. Fadeev, A.L. Goldstein, I. Grevtsev, G. Hirschhorn, I. Jeroukhimov, A. Kedar, Y. Klein, A. Korin, B. Levit, I. Schrier, A.D. Schwarz, W. Shomar, D. Soffer, M. Weiss, O. Yaslowitz, I. Zoarets, Gilad Twig, Mor Rittblat, Shahar Shapira

**Affiliations:** 1 Israeli Defense Forces, Medical Corps, Tel Hashomer, Ramat Gan, Israel; 2 Department of Otolaryngology-Head and Neck Surgery, Rabin Medical Center, Petah Tikva, Israel; 3 Department of Military Medicine and “Tzameret,” Faculty of Medicine, The Hebrew University of Jerusalem, Jerusalem, Israel; 4 Division of Anesthesia, Intensive Care & Pain Management, Tel-Aviv Sourasky Medical Center, Tel-Aviv, Israel; 5 Gertner Institute of Epidemiology and Health Policy Research, Sheba Medical Center, Ramat Gan, Israel; 6 Faculty of Medicine, Tel Aviv University, Tel Aviv, Israel; 7 Arrow Program for Medical Research Education, Sheba Medical Center, Ramat Gan, Israel; 8 Department of Plastic and Reconstructive Surgery, Hadassah Hebrew University Medical Centre, Jerusalem, Israel

**Keywords:** combat-related neck trauma, penetrating injuries, military conflict epidemiology, surgical exploration, trauma registry analysis, ICU admission and outcomes, rapid intervention strategies

## Abstract

**Objectives:**

Neck injuries in warfare are critical due to the concentration of vital structures within a relatively unprotected anatomical region. This study aims to provide a comprehensive analysis of the epidemiology, severity, and outcomes of neck injuries during Military operations under “Operation Swords of War,” leveraging data from the Israeli National Trauma Registry.

**Methods:**

Data were retrospectively collected for casualties from October 7, 2023, through December 31, 2023. Injury characteristics, time to surgical exploration, and in-hospital outcomes were analyzed.

**Results:**

A total of 1815 trauma casualties were recorded, with 147 (8.1%) suffering from neck injuries. The majority of these injuries were due to penetrating trauma (89.8%), with a significant number leading to surgical exploration (45.0%). The study highlighted the extensive use of resources for managing these severe injuries, including operating rooms and intensive care units.

**Conclusion:**

The findings underscore the high prevalence and severity of neck injuries in military conflicts and the critical need for rapid assessment and intervention. Comparisons with previous conflicts suggest an improvement in survival rates due to enhanced medical response and triage efficiency. In conclusion, neck injuries remain a significant concern in combat settings, necessitating specialized trauma care and rapid intervention strategies. The results from this study provide vital insights that can help improve trauma care protocols and outcomes in future conflicts.

**The Bottom Line**

Neck injuries in combat are dangerous but understudied. This analysis of over 1800 trauma patients injured during the 2023 Military operations under “Operation Swords of War” found that 8.1 percent suffered neck injuries—most from penetrating trauma. Nearly half required surgery, and many needed intensive care. Using data from the Israeli National Trauma Registry, the study highlights how these injuries demand rapid, resource-intensive care. Compared with previous wars, survival appears to be improving, likely due to faster triage and better frontline care. These findings help refine trauma response protocols for future military and civilian mass casualty situations.

## 1 Introduction

### 1.1 Background

Although the neck represents 1% of the total body surface, it disproportionately accounts for 16% to 39% of wartime injuries in conflicts studied from 1914 to 2007,^1^^,^^2^ with prevalence escalating in recent conflicts. The neck contains the following multiple vital structures: major blood vessels, nerves, and the upper aerodigestive tract. Moreover, although the neck houses these structures, its anatomical structure lacks natural shielding, unlike the head or the chest. As a result, penetrating or blunt neck injuries have devastating consequences and are highly fatal.^3^^,^^4^ Low-energy injuries can also be harmful, and studies have attributed neck wounds as the primary cause of death in 41% to 73% of service personnel.^5^ Additionally, nonfatal injuries may inflict substantial disability and prematurely end the operational careers of service personnel.^4^

Historical evidence demonstrates the high incidence of head, face, and neck injuries among battlefield injuries.^4^^,^^6^^,^^7^ However, the shift toward the use of fragmentary blast devices and the reduction in mortality from head and neck trauma in recent conflicts, attributed mainly to advanced medical interventions at the point of injury and expedited military evacuation strategies, have significantly improved survival rates from such devastating injuries.^5^^,^^8^ Prehospital identification and treatment of neck injuries can be challenging, as advanced life support providers have limited tools to evaluate the extent of the injury and the involvement of vital structures at the point of injury.^9^ Thus, there is a substantial risk of underestimation of neck injury severity.^10^

Historically, mandatory neck exploration was the standard, but it often resulted in a high rate of nontherapeutic surgeries. However, recent studies emphasize the importance of adopting a selective approach to surgical exploration guided by clinical criteria and advanced imaging techniques. This approach significantly reduces unnecessary explorations without compromising patient safety.^11^ Selective management relies on clinical examination and adjunctive tests such as angiography and endoscopy to identify life-threatening injuries.^12^ Despite its challenges, neck exploration remains indispensable for casualties showing clear signs of vascular or aerodigestive tract injuries.^13^

### 1.2 Importance

Since the start of the armed conflict between Israel and Hamas, known as the Swords of Iron War (SOI), on October 7, 2023, the Israeli health care system has faced various challenges, including mass casualty events and the day-to-day inflow of casualties from the war zones.

### 1.3 Goals of This Investigation

This study aims to document the incidence, severity, and outcomes of neck injuries among civilians and military personnel during the Israeli-Hamas armed conflict.

## 2 Methods

### 2.1 Ethical Considerations

The study was approved by the Sheba Medical Center Institutional Review Board (SMC 5138-18). The manuscript was written and edited according to the Strengthening the Reporting of Observational Studies in Epidemiology (STROBE) statement guidelines.^14^

### 2.2 Study Design and Setting

This current study extracted data from hospitalized Israeli trauma casualties recorded in the Israeli National Trauma Registry (INTR) due to the Israeli-Hamas armed conflict. We included incidents occurring between October 7 (the date the war was declared) and December 31, 2023.

### 2.3 Participants

This study included adult trauma casualties (aged >18 years) who were hospitalized following injury, between October 7 and December 31, 2023. Only patients admitted to hospitals and recorded in the INTR were eligible for inclusion. Casualties who were declared dead upon hospital arrival or lacked documented injury characteristics were excluded. Additionally, nonhospitalized casualties were excluded due to the unavailability of in-hospital data.

### 2.4 Data Extraction

The INTR exclusively includes inpatients, defined as trauma casualties admitted to the hospital. It is a nationwide database encompassing all 7 level-I trauma centers and 16 level-II trauma centers, ensuring comprehensive coverage of hospitalized Israeli-Hamas armed conflict casualties.^15^ It includes patients diagnosed with trauma-related injuries, as defined by the International Classification of Diseases, Ninth Revision, Clinical Modification codes 800-959.9. Although it captures cases of patients who died in the emergency department (ED) or were transferred between facilities, it excludes those who died before reaching the hospital, were discharged after ED treatment, or were admitted more than 72 hours postinjury. Cases involving poisoning, drowning, or suffocation are also omitted. Data quality is rigorously maintained through systematic recording by trained trauma registrars at each participating center, under the supervision of designated trauma directors and coordinators. Electronic records undergo comprehensive quality checks before inclusion in the registry and subsequent analysis.

### 2.5 Variables

The extracted data included the following variables: date of injury, demographic characteristics, vital signs, and clinical information, such as injury etiology, severity, diagnoses, surgical procedures, intensive care unit (ICU) admission, hospital length of stay, and discharge destination. Diagnoses were classified using the abbreviated injury scale, which categorizes injury severity as follows: 1, minor, 2, moderate, 3, serious, 4, severe, 5, critical, and 6, unsurvivable. Additionally, we systematically analyzed the time elapsed from the patient’s arrival at the medical facility to the initiation of exploratory neck surgery. Time intervals were categorized into 5 distinct groups to facilitate a detailed assessment of the timing of surgical interventions.

### 2.6 Statistical Analysis

Statistical analysis included chi-square tests and Fisher exact tests for categoric variables such as the following: date of injury, population group, sex, mechanism of injury, injury severity score (ISS), and ICU admission. Those are presented as frequencies and percentages. Age, as a continuous variable, is represented by medians and interquartile ranges and analyzed using Kruskal-Wallis tests. All statistical analyses were performed using the SAS Software version 9.4 (SAS). A *P* value less than .05 was considered statistically significant.

## 3 Results

### 3.1 Baseline Injury Characteristics and In-Hospital Measures and Outcomes

Our cohort included 1815 injured individuals, comprising 1668 (91.9%) without neck injuries and 147 (8.1%) with neck injuries. Significant differences were noted between these 2 groups. Casualties with neck injuries had a median age of 24 years (range: 18-68), whereas those without neck injuries had a median age of 25 years (range: 0-97). The neck injury group was predominantly soldiers (126 of 147, 85.7% vs 1118 of 1668, 67.0%; *P* < .01). ISS was higher for neck injuries, with more cases scoring 25+, as well as the prevalence of penetrating injuries (24 of 147, 16.3% vs 146 of 1668, 8.7%; 132 of 147, 89.8% vs 1249 of 1668, 74.9%; accordingly, *P* < .01). Computed tomography scans were performed in the emergency room more frequently among casualties with neck injuries (109 of 147, 74.2% vs 911 of 1668, 54.6%; *P* < .01). In addition, the ED disposition and ICU admission showed significant differences between casualties with and without neck injuries. Casualties with neck injuries were more often directed to the operating room and the ICU (46 of 147, 31.3% vs 370 of 1668, 22.2%; 14 of 147, 9.5% vs 89 of 1668, 5.3%; accordingly, *P* =.01). For other characteristics, including surgery requirements, length of stay, and discharge destination, refer to Tables 1 and 2.

**Table 1:** Comparative analysis of neck injuries versus non-neck injuries from military operations under “Operation Swords of War.”

Variable	No neck injury (N = 1668), n (91.9%)	Neck injury (N = 147), n (8.1%)	*P* value^a^
Date of injury			.03
07-08 October, 2023	661 (39.6%)	45 (30.6%)	
09 October, 2023 to 31 December, 2023	1007 (60.4%)	102 (69.4%)	
Population group			<.01
Soldiers	1118 (67.0%)	126 (85.7%)	
Civilians	550 (33.0%)	21 (14.3%)	
Gender			<.01
Male	1437 (86.1%)	142 (96.6%)	
Female	231 (13.9%)	5 (3.4%)	
Age (y) (median [range])	25 [0-97]	24 [18-68]	<.01
Mechanism of injury			<.01
Explosion	655 (39.3%)	86 (58.5%)	
Gunshot wound	665 (39.9%)	51 (34.7%)	
Explosion + gunshot wound	31 (1.8%)	2 (1.4%)	
Other	317 (19.0%)	8 (5.4%)	
Penetrating injury, Yes	1249 (74.9%)	132 (89.8%)	<.01
Associated injuries (AIS 3+), Yes			
Head	133 (8.0%)	11 (7.5%)	.83
Face	36 (2.2%)	8 (5.4%)	.01
Chest	208 (12.5%)	27 (18.4%)	.04
Abdomen	138 (8.3%)	6 (4.1%)	.07
Spine	29 (1.7%)	0 (0.0%)	<.01
Upper extremities	160 (9.6%)	15 (10.2%)	.81
Lower extremities	296 (17.8%)	17 (11.6%)	<.01
Injury severity score			<.01
1-8	896 (53.8%)	85 (57.8%)	
9-14	416 (25.0%)	21 (14.3%)	
16-24	208 (12.5%)	17 (11.6%)	
25+	146 (8.7%)	24 (16.3%)	
Glasgow coma scale			.25
3-8	68 (4.2%)	10 (7.0%)	
9-14	48 (3.0%)	3 (2.1%)	
15	1495 (92.8%)	129 (90.8%)	
ER disposition			<.01
Operating room	370 (22.2%)	46 (31.3%)	
ICU	89 (5.3%)	14 (9.5%)	
Ward	1199 (71.9%)	86 (58.5%)	
Death	10 (0.6%)	1 (0.7%)	
CT performed in ER, Yes	911 (54.6%)	109 (74.2%)	<.01
ICU admission, Yes	237 (14.2%)	37 (25.2%)	<.01
Surgery required			
Yes	963 (57.7%)	83 (56.5%)	
No	705 (42.3%)	64 (43.5%)	
Length of stay (d)			<.01
0-1	454 (27.2%)	47 (32.0%)	
2-6	710 (42.6%)	55 (37.4%)	
7-13	238 (14.3%)	14 (9.5%)	
14+	264 (15.9%)	31 (21.1%)	
Discharge destination			<.57
Home	1154 (69.2%)	106 (72.1%)	
Rehabilitation	458 (27.5%)	38 (25.9%)	
Death	37 (3.3%)	3 (2.0%)	

AIS, abbreviated injury scale; ER, emergency room; CT, computed tomography; ICU, intensive care unit.

a *P* values acquired from chi-squared test, Fisher's Exact test or Kruskal-Wallis test.

**Table 2:** Comparative analysis neck injuries requiring neck exploration versus those not requiring exploration from military operations under “Operation Swords of War.”

Variable	Neck exploration (N = 66), n (44.9%)	No neck exploration (n = 81), n (55.1%)	*P* value^a^
Date of injury			.17
07-08 October, 2023	24 (36.4%)	21 (25.9%)	
09 October, 2023 to 31 October, 2023	42 (63.6%)	60 (74.1%)	
Population			.46
Soldiers	55 (83.3%)	71 (87.7%)	
Civilians	11 (16.7%)	10 (12.3%)	
Gender			.82
Male	64 (97.0%)	78 (96.3%)	
Female	2 (3.0%)	3 (3.7%)	
Age (y) (median [range])	24.5 [19-52]	23 [18-68]	.11
Mechanism of injury			.13
Explosion	35 (53.0%)	51 (63.0%)	
Gunshot wound	27 (40.9%)	24 (29.6%)	
Explosion + gunshot wound	2 (3.0%)	0 (0.0%)	
Other	3 (4.5%)	6 (7.4%)	
Penetrating injury			.34
Yes	61 (92.4%)	71 (87.7%)	
Injury severity score			.02
1-8	29 (43.9%)	56 (69.1%)	
9-14	12 (18.2%)	9 (11.1%)	
16-24	9 (13.6%)	8 (9.9%)	
25+	16 (24.2%)	8 (9.9%)	
Glasgow coma scale			.23
3-8	7 (11.1%)	3 (3.8%)	
9-14	1 (1.6%)	2 (2.5%)	
15	55 (87.3%)	74 (93.7%)	
CT performed in ER, Yes	45 (68.2%)	64 (79.0%)	.14
Vascular injury, Yes	10 (15.2%)	4 (4.9%)	.04
Associated injuries (AIS 3+)			
Head	6 (9.1%)	5 (6.2%)	.50
Face	6 (9.1%)	2 (2.5%)	.08
Chest	16 (24.2%)	11 (13.6%)	.10
Abdomen	6 (9.1%)	0 (0%)	<.01
Spine	4 (6.1%)	4 (4.9%)	.77
Upper extremities	12 (18.2%)	3 (3.7%)	<.01
Lower extremities	8 (12.1%)	1 (1.2%)	<.01
Hospitalization location			<.01
Operating room	37 (56.1%)	9 (11.1%)	
Intensive care unit	6 (9.1%)	8 (8.1%)	
Surgical ward	23 (34.8%)	63 (80.8%)	
Presence of other injuries			<.01
Yes	39 (59.1%)	17 (21.0%)	
No	27 (40.9%)	64 (79.0%)	
Discharge outcome			.02
Death	2 (3.0%)	1 (1.2%)	
Home	40 (60.6%)	66 (81.5%)	
Rehabilitation	24 (36.4%)	14 (17.3%)	

AIS, abbreviated injury scale; CT, computed tomography; ICU, intensive care unit.

a *P* values acquired from chi-squared test, Fisher's Exact test or Kruskal-Wallis test.

### 3.2 Neck Injuries Necessitating Exploration

Table 2 categorizes the injury characteristics and outcomes of the 147 casualties with neck injuries based on whether they underwent surgical neck exploration. Among these, 66 (44.9%) had explorations, displaying notably different outcomes compared with the 81 (55.1%) who did not. Specifically, those who underwent exploration were more likely to have vascular injuries (15.0% vs 5.0%; *P* =.04) and exhibited significantly higher heart rates of 130+ bpm (5.4% vs 0%; *P* =.04).

The clinical outcomes were notably different between these 2 groups. Casualties requiring neck exploration were more often referred to the operating room directly from the emergency room (37 of 66, 56.1% vs 9 of 81, 11.1%) and experienced a greater need for ICU care (24 of 66, 36.4% vs 13 of 81, 16.0%; *P* < .01). Hospital stays were generally longer for these casualties, with a higher proportion staying ≥14 days (21 of 66, 31.8% vs 10 of 81, 12.3%; *P* < .01). Discharge outcomes also showed significant differences, with neck exploration casualties more often discharged to rehabilitation (24 of 66, 36.4% vs 14 of 81, 17.3%; *P* = .02). For further details, refer to Table 2.

**Figure 1:**
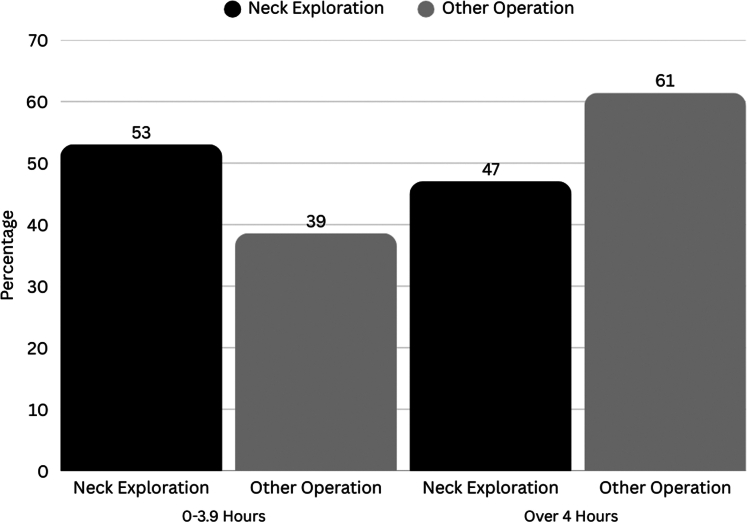
Divided percentage of time to neck exploration versus other procedures. The graph displays the divided percentage of cases explored over time, segmented into the 2 intervals: 0-3.9 hours, and 4+ hours. Each sky-blue bar represents the percent of explorations completed at the interval.

Figure 1 describes the analysis of the time from patient arrival to the initiation of exploratory neck surgery, revealing significant variations in the timing of surgical intervention. Within the first 4 hours of arrival, 53.0% of patients underwent neck exploration compared with only 38.6% of patients who underwent other procedures (*P* = .02).

**Figure 2:**
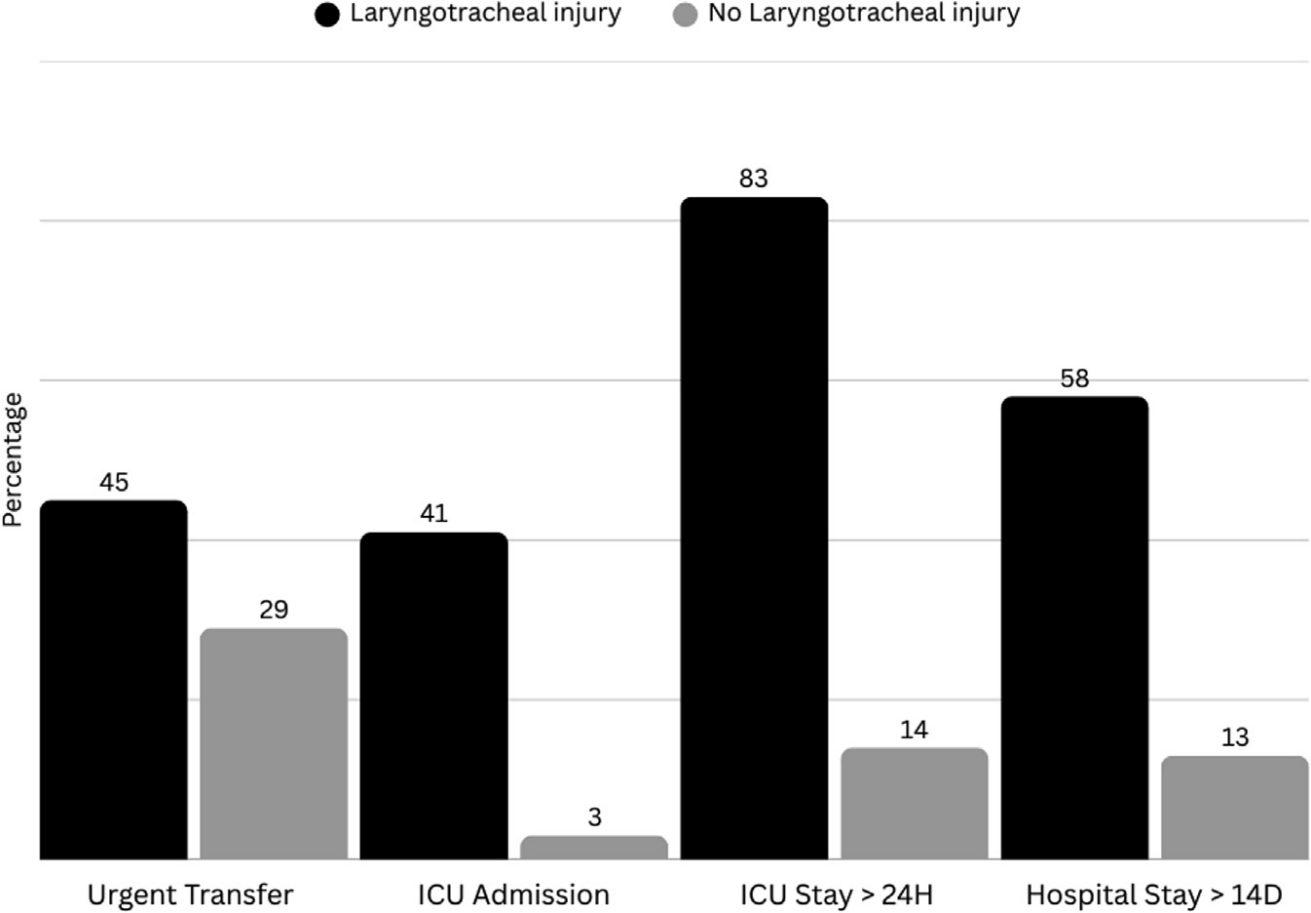
Emergency referral and hospitalization distribution in patients with laryngotracheal injuries. The graph displays the comparative distribution of clinical outcomes between patients with vascular injuries and those without vascular injuries. Each blue bar represents the percentage of patients for each variable, illustrating the referral and hospitalization distribution

**Figure 3:**
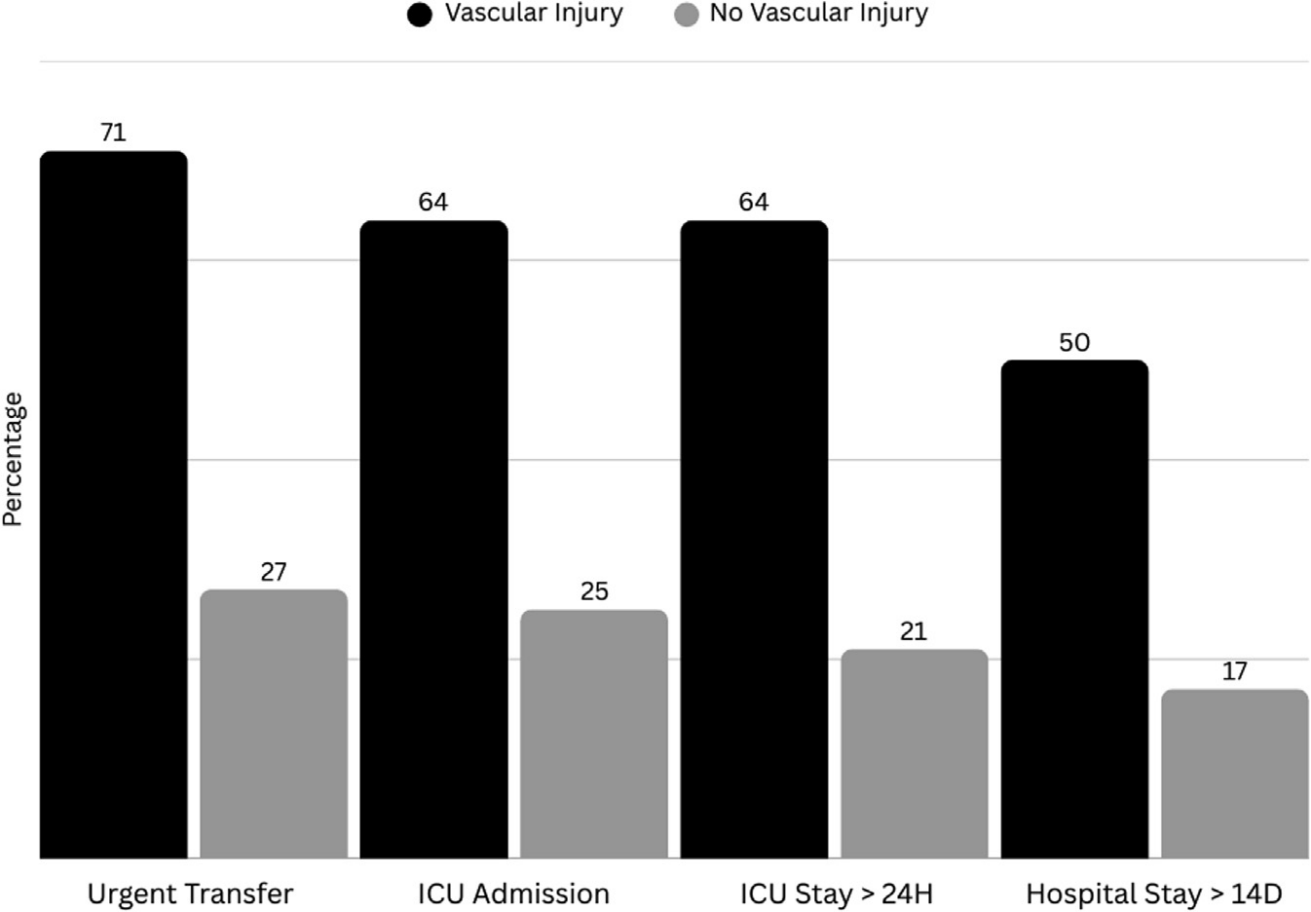
Emergency referral and hospitalization distribution in patients with vascular injury. The graph displays the comparative distribution of clinical outcomes between patients with laryngotracheal injuries and those without laryngotracheal injuries. Each blue bar represents the percentage of patients for each variable, illustrating the referral and hospitalization distribution.

Figures 2 and 3 show subanalyses for patients with laryngotracheal and vascular injuries; patients with laryngotracheal injuries were more likely to be urgently transferred to the operating room (45.8%) compared with their noninjured counterparts (28.5%), with a statistically significant difference (*P* < .0001). Similarly, the presence of vascular injuries significantly increased the likelihood of urgent operating room (OR) transfer (71.4% vs 27.1%, *P* < .0001). ICU admission rates were markedly higher among patients with laryngotracheal (41.7% vs 3.3%, *P* < .0001) and vascular injuries (64.3% vs 25.5%, *P* < .0001). Furthermore, patients with these injuries experienced longer ICU stays and hospitalizations.

## 4 Limitations

This study is subject to several significant limitations. First, a retrospective analysis based on a national trauma registry may be affected by variability across participating centers and potential inaccuracies in data entry despite quality assurance efforts to standardize data. Second, the registry does not account for nonhospitalized casualties, including those who may die en route or are discharged from the ED due to minor injuries. Consequently, the study might overestimate the true scope and severity of neck injuries sustained during the Military operations under the Israeli-Hamas armed conflict.

Additionally, the registry lacks detailed data on the specific regions of neck injuries and critical aspects like vascular and neurologic compromise, limiting the scope of our findings. Despite these limitations, the study provides valuable insights that can inform the epidemiology of neck injuries in similar war zones and among civilian populations. Future research should focus on assessing specific neck injury outcomes, long-term complications, surgical interventions, and the associated economic impacts. Such studies will enhance our understanding of the overall consequences of primary neck injuries. Moreover, a detailed examination of mechanism of injury and locations will help evaluate the feasibility of neck collar protection in the unique context of urban warfare in the Middle East.

## 5 Discussion

Using national registry data, we characterized the epidemiology and clinical impact of neck injuries during military operations under the Israeli-Hamas armed conflict. These injuries were primarily penetrating, with elevated ISS scores, higher ICU admission rates, and frequent need for emergency surgical intervention. Exploration was also linked to increased discharge to rehabilitation facilities, reflecting the long-term functional burden of severe neck trauma.

The observed neck injury rate (8.1%) is consistent with previous reports from conflict zones, such as the US and UK military cohorts, where rates ranged from 11% to 19%.^5^^,^^6^ The Israel Defense Forces Medical Corps holds unpublished data showing similar findings, according to which 20% and 11% of the casualties suffered from head and neck injuries during the Yom Kippur War (1973) and the First Lebanon War (1982), respectively.^7^

Consistent with prior studies, the predominance of penetrating trauma (89.8%) underscores the neck’s anatomical vulnerability in modern warfare.^11^^,^^16^^,^^17^ Moreover, studies report that up to 87% of explorations in actively bleeding casualties confirm critical injuries, underscoring the necessity of exploration in select cases.^12^^,^^18^ Surgical exploration was required in 45% of cases, particularly those with hemodynamic instability or vascular injury, echoing prior evidence supporting early intervention in selected patients.^19^^,^^20^

Multiple clinical markers reflected that neck injuries were associated with high severity, hemodynamic instability, ICU admission (25.2%), and emergent OR referral (31.3%), collectively illustrating the critical nature of neck injuries in our cohort. Furthermore, neck injuries were associated with higher ISS, hypotension, and a greater need for critical care, consistent with their high clinical severity.

These findings are consistent with previous studies, which observed that neck injuries from penetrating trauma are often life-threatening and require immediate surgical management.^20^, ^21^, ^22^ The high rate of computed tomography use (74.2%) underscores the difficulty of relying solely on physical examination to assess neck trauma, reaffirming the central role of imaging in modern triage. Subgroup analysis demonstrated that laryngotracheal and vascular injuries were strongly associated with urgent OR referral and ICU admission, reinforcing their prognostic significance. The significantly higher rates of urgent transfers to the OR and ICU admissions for patients with these injuries suggest a heightened risk of severe complications and the need for immediate medical intervention.^23^ The prolonged ICU stays and extended hospitalizations not only reflect the severity of the injuries but also point to the substantial health care resources required for these patients.^24^

The Israeli health care system faced, in the 24 hours of October 7, a mass casualty event, potentially limiting access to prompt and adequate prehospital treatment for all injured individuals. Data from the INTR indicate that during the initial 24 hours of the conflict, emergency services were mobilized to manage over 300 critically wounded individuals, stretching resources thin and possibly affecting the immediate care available for neck trauma casualties.^25^^,^^26^ Nonetheless, improved survivability may reflect system-wide advances in trauma response and evacuation.^27^, ^28^, ^29^ Although prior studies recommend early exploration (<2 hours), our data show substantial variation, suggesting real-world delays due to triage, diagnostics, or logistical constraints.^30^, ^31^, ^32^

In summary, our findings provide an overview of neck injury admissions in Israel during Military operations under the Israeli-Hamas armed conflict, emphasizing the severity and urgency of combat-related neck injuries and highlighting the importance of early intervention and targeted trauma care protocols.

## Author Contributions

NT: Data curation, investigation, methodology, project administration, writing–original draft and writing–review and editing.

MR: Data curation, Conceptualization, methodology, resources, writing–original draft and editing.

TT: Investigation, Formal analysis, methodology, writing–review and editing.

IR: Conceptualization, Data curation, investigation, formal analysis, and methodology.

IR: Data curation, investigation, Formal analysis, methodology.

AG: Visualization, Data curation, investigation, Formal analysis, methodology and writing–review and editing.

SS: Conceptualization, methodology, supervision, resources, and writing–review and editing.

GT: Conceptualization, methodology, resources, supervision, resources, writing–review and editing.

## Funding and Support

By *JACEP Open* policy, all authors are required to disclose any and all commercial, financial, and other relationships in any way related to the subject of this article as per ICMJE conflict of interest guidelines (see www.icmje.org). The authors have stated that no such relationships exist.

## Conflict of Interest

All authors have affirmed they have no conflicts of interest to declare.
